# Supplementary material for: AAV2retro Enters Axons of Passage and Extensively Transduces Corticospinal Neurons After Injection into Spinal White Matter
Source: Brain Sci. 2025 Sep 28;15(10):1058. doi: 10.3390/brainsci15101058 (PMC12564753; doi:10.3390/brainsci15101058)
Supplement: Supplementary file 1 [file brainsci-15-01058-s001.zip › brainsci-3761343-supplementary material 25-09-29.pdf]

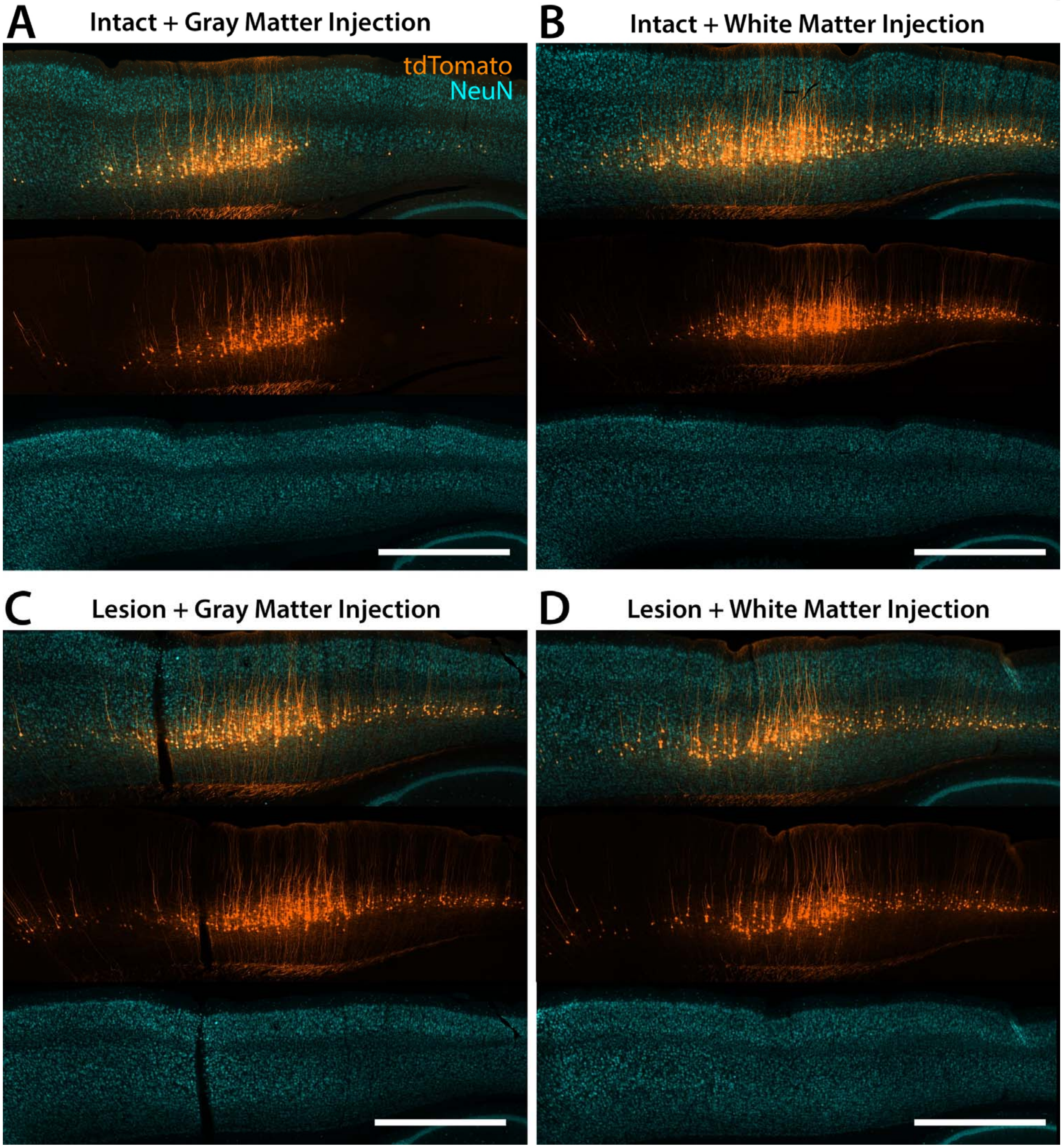

Supplementary Figure S1. Transduced neurons in sensorimotor cortex are confined to layer 5 after injection of AAV2retro into C4 spinal cord. For each group, the medial section with the largest number of tdTomato-labeled neurons is shown (quantified in Figure 2A). Immunolabeling of tdTomato (orange) and NeuN (blue) demonstrates that transduced neurons are confined to layer 5 in all groups, with no detectable labeling of cells in other cortical layers, consistent with specific gene delivery to layer 5 corticospinal projection neurons by retrograde transport. All sections were stained and imaged in parallel under identical conditions. Adjustments to improve visibility were applied identically to all images. Scale bars: 1 mm.

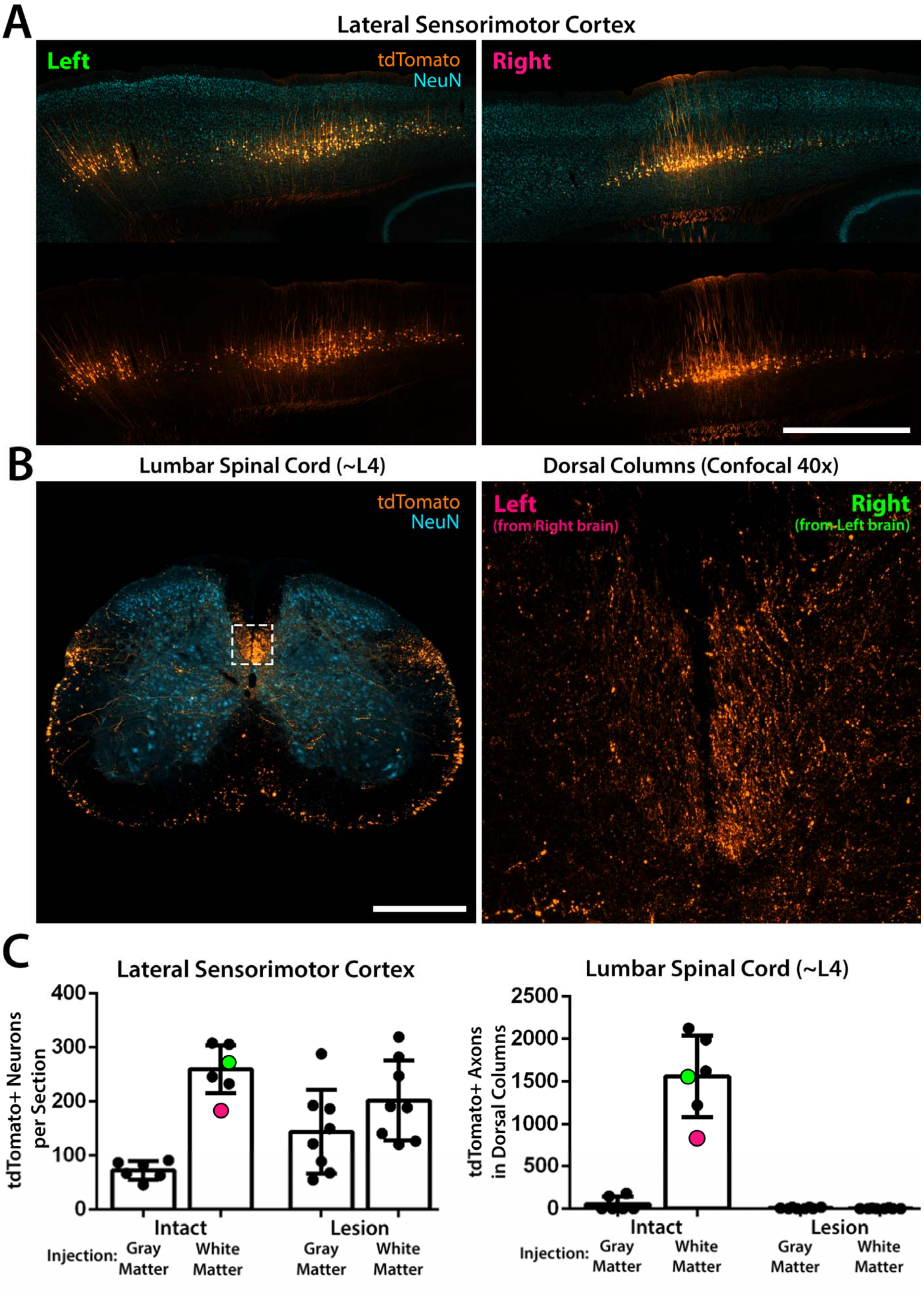

Supplementary Figure S2. Efficacy of gene delivery to corticospinal neurons can vary between the left and right sides within the same animal. (**A**) In an animal treated by injection of AAV2retro into intact C4 dorsal column white matter, the left and right sides of the brain show differences in the

number and distribution of tdTomato-labeled corticospinal neurons in lateral sensorimotor cortex (1.7-2.0 mm from midline). Scale bar: 1 mm. (**B**) In L4 lumbar spinal cord from the same animal as panel A, more tdTomato-labeled corticospinal axons are visible in the right corticospinal tract (descending from the left side of the brain) than in the left corticospinal tract (descending from the right side of the brain), consistent with the differences observed in sensorimotor cortex. Scale bar: 0.5 mm. (**C**) Quantification of tdTomato-labeled neurons in lateral sensorimotor cortex and tdTomato-labeled axons in lumbar spinal cord (reproduced from Figures 2 and 3). The left brain and right corticospinal tract (descending from the left brain) are highlighted in green. The right brain and left corticospinal tract (descending from the right brain) are highlighted in pink. Error bars indicate standard deviation. The differences observed between the left and the right corticospinal tracts within the same animal support the anatomical analysis of each side as an independent circuit. All sections were stained and imaged in parallel under identical conditions. Adjustments to improve visibility were applied identically to all images.

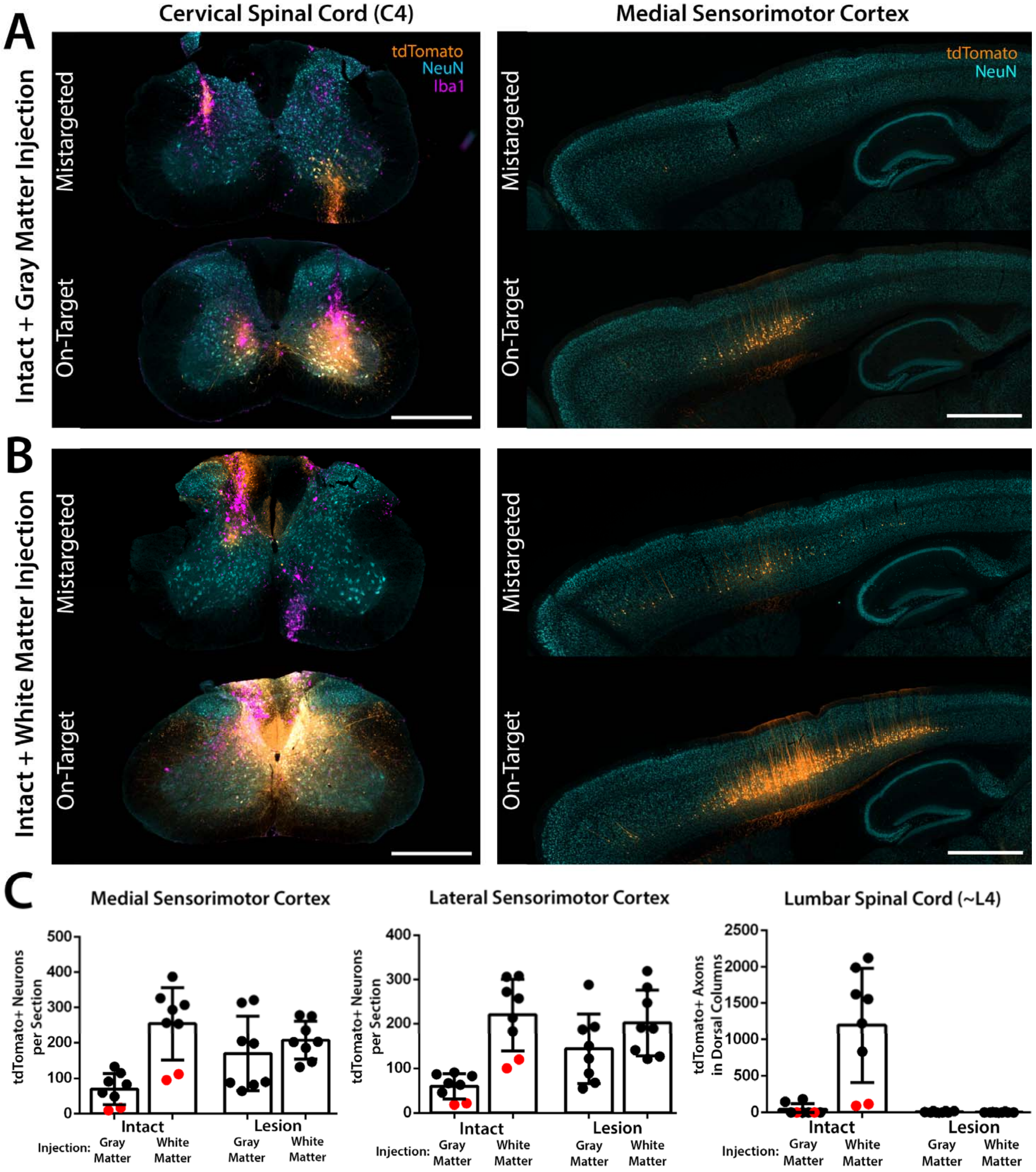

Supplementary Figure S3. Mistargeting of AAV2retro injections greatly reduces transduction of corticospinal neurons. (**A**) In one animal that received injections of AAV2retro targeting the intact gray matter, injections were too deep (mistargeted to the ventral white matter), resulting in visibly reduced transduction of corticospinal neurons in medial sensorimotor cortex. (**B**) In one animal that received injections of AAV2retro targeting the intact white matter, injections were too lateral (mistargeted to the dorsal gray matter), resulting in visibly reduced transduction of corticospinal neurons in medial sensorimotor cortex. (**C**) Quantification of tdTomato-labeled neurons in sensorimotor cortex and tdTomato-labeled axons in lumbar spinal cord, reproduced from Figures 2 and 3 with the mistargeted injections included (highlighted in red). Error bars indicate standard deviation. For all analyses, every

mistargeted injection fell more than one standard deviation below the mean (except axon count in lumbar spinal cord after intact gray matter injection, for which both mistargeted datapoints were equal to zero). This supports the exclusion of these mistargeted injections from further analysis. All sections were stained and imaged in parallel under identical conditions. Adjustments to improve visibility were applied identically to all images.
